# An efficient algorithm for solving piecewise-smooth dynamical systems

**DOI:** 10.1007/s11075-021-01154-1

**Published:** 2021-07-05

**Authors:** Nicola Guglielmi, Ernst Hairer

**Affiliations:** 1 Gran Sasso Science Institute, via Crispi 7, I-67100 L’Aquila, Italy; 2 Section de Mathématiques, Université de Genève, rue du Conseil-Général 7-9, CH-1205 Genève, Switzerland

**Keywords:** Piecewise-smooth systems, Filippov solution, Codimension-2 manifold, Regularization, Hidden dynamics, Scaling invariance, 34A36, 34A60, 65L05, 65L80

## Abstract

This article considers the numerical treatment of piecewise-smooth dynamical systems. Classical solutions as well as sliding modes up to codimension-2 are treated. An algorithm is presented that, in the case of non-uniqueness, selects a solution that is the formal limit solution of a regularized problem. The numerical solution of a regularized differential equation, which creates stiffness and often also high oscillations, is avoided.

## Introduction

Piecewise-smooth dynamical systems arise in many applications and they are an active field of recent research. Historically, one of the first examples is Coulomb friction in mechanical systems, where the force of friction is proportional to the sign of velocity (see [5]). Many interesting applications can be found in the monograph [6]: relay control systems, where the control variable admits jump discontinuities; converter circuits, where switching devices lead to a non-smooth dynamics; models in the social and financial sciences, where continuous change can trigger discrete actions. Discontinuity points are also created by the activation/deactivation of inequality constraints in mixed constrained optimization problems. See [24] for a particular application arising in the modelling of atmospheric particles.

For a mathematical formulation of the problem we consider discontinuity hyper-surfaces

1.1 $$ {{\varSigma}}_{j} = \{ y \in{\mathbb{R}}^{n} | \alpha_{j} (y) =0 \} ,\qquad j=1, {\ldots} ,d, $$

where $\alpha : {\mathbb {R}}^{n} \to {\mathbb {R}}^{d}$ (with *d* < *n*) is assumed to be sufficiently differentiable and such that these hyper-surfaces intersect transversally. We denote the discontinuity set by ${{\varSigma }} = \bigcup _{j=1}^{d} {{\varSigma }}_{j}$. The hyper-surfaces *Σ*_*j*_ divide the phase space ${\mathbb {R}}^{d}\setminus {{\varSigma }}$ into 2^*d*^ open regions

1.2 $$ {\mathcal{R}}^{{\mathbf k}} = \big\{ y \in{\mathbb{R}}^{n} \big| k_{j} \alpha_{j} (y) > 0 ~\text{for}~ j=1, {\ldots} ,d \big\} , $$

where **k** = (*k*_1_,…,*k*_*d*_) is a multi-index with *k*_*j*_ ∈ {− 1, 1}. The discontinuous dynamical system is then given by

1.3 $$ \dot y = f^{{\mathbf k}} (y) \qquad\text{for}\qquad y \in {\mathcal{R}}^{{\mathbf k}} . $$

We assume that the functions *f*^**k**^(*y*) are defined in a neighbourhood of the closure of $ {\mathcal {R}}^{{\mathbf k}}$ and that they are sufficiently differentiable. In the discontinuity set *Σ* the right-hand side of (1.3) is considered to be multi-valued with values from the neighbouring domains. We are thus concerned with a differential inclusion and we adopt a restriction of the approach by Filippov [13, 14] for the concept of solutions. Besides classical solutions, which cross the discontinuity surfaces, there are also sliding modes evolving in the discontinuity set *Σ*.

Closely connected to a discontinuous dynamical system is a regularization, where the jump discontinuities are replaced in an *ε*-neighbourhood by a continuous transition. In this way the differential inclusion is transferred to an ordinary differential equation. It is natural to consider regularizations because, as mentioned in [6, p. 1], “… there is strictly speaking no such thing as a piecewise-smooth dynamical system and that in reality all physical systems are smooth”. This is precisely what happens in the analysis of gene regulatory networks [12, 26], where steep sigmoid-type nonlinearities are approximated by step functions.

Among numerically sound approaches for approximating the solution of (1.3) let us mention the following two:

- *Algorithm based on event detection.* One locates accurately the time instants when the solution enters a new discontinuity surface (or satisfies a criterion for exiting a surface), one stops the integration and investigates the possible solutions leaving the actual point, and then one continues the integration with a new vector field. The disadvantage of this approach is that at the actual point the discontinuous problem can have more than one solution (sometimes even infinitely many), and it may be laborious to follow all of them.
- *Regularization.* One solves numerically the regularized ordinary differential equation, which provides a unique approximation. Here, the difficulty is the choice of the regularization parameter *ε* > 0. To obtain a good approximation of the solution of (1.3) a very small *ε* is required. This implies that the regularized differential equation is stiff and sometimes highly oscillatory, so that the numerical integration may become expensive.

Early work on solving piecewise-smooth dynamical systems that is based on detecting, locating, and passing the discontinuity is published in [4, 15, 27]. For a survey we refer to [10]. There are some recent publications (including Matlab codes for solving piecewise-smooth dynamical systems), like those of [29] and [3], that are reliable and carefully compute the switching points between classical and Filippov solutions. All these publications are restricted to classical solutions and to sliding modes in codimension 1. Our main interest is the situation, where codimension-2 sliding modes can occur.

In the present work we propose an algorithm that combines the advantages of both approaches. With event detection we solve the discontinuous problem (without any *ε*) but, instead of following all solutions in the case of non-uniqueness, we propose to select the solution which can formally be interpreted as the limit solution (for *ε* → 0) of a regularized differential equation. This selection is partly done on the basis of the classification in [17].

In Section 2 we recall concepts needed for the understanding of the present article (relation between sliding modes and differential-algebraic equations of index 2, regularization, hidden dynamics, and scaling invariance). The structure of the algorithm for solving the discontinuous system (1.3) is given in Section 3. The main part (Section 4) presents in an algorithmic way the switching between different kinds of solutions at the discontinuity hyper-surfaces. This part is independent of the regularization, in contrast to Section 5, where a justification of the algorithm (based on the hidden dynamics) is given. The article finishes with some comments on the implementation (Section 6) and a conclusion (Section 7).

## Solution concept and regularization

The definition of Filippov solutions for a discontinuous dynamical system (1.3) is ambiguous, because in the intersection of discontinuity hyper-surfaces a convex combination of the adjacent vector fields has too many degrees of freedom. We restrict our study to special convex combinations having *m* parameters in the intersection of *m* hyper-surfaces *Σ*_*j*_. Such convex combinations (for *m* = 2) are called “blending” in [1] and “bilinear interpolation” in [7, 8], see also [9, 25]. For arbitrary *m* they are called “convex canopy” in [20]. We consider regularizations that are closely connected to such convex combinations, and we call them “multi-linear interpolation”.

### Solution concept — classical solutions and sliding modes

For a fixed multi-index **k** = (*k*_1_,…,*k*_*d*_) with *k*_*j*_ ∈ {− 1, 1} the equation (1.3) is a regular ordinary differential equation on the open domain ${\mathcal {R}}^{{\mathbf k}}$, and the standard theory on existence, uniqueness, and continuous dependence on parameters and initial values applies. In this case the solution of (1.3) is called *classical*.

We next extend the concept of solution to the discontinuity set *Σ*. For an index vector **k** = (*k*_1_,…,*k*_*d*_) with *k*_*j*_ ∈ {− 1, 0,1} (note that now *k*_*j*_ can also be zero) we consider the set

2.1 $$ {\mathcal{R}}^{{\mathbf k}} = \Big\{ y \in{\mathbb{R}}^{n} \big| \alpha_{j} (y) = 0 ~\text{if}~k_{j} =0,~ k_{j}\alpha_{j}(y) >0 ~\text{if}~k_{j} \ne0 \Big\} , $$

and if at least one component *k*_*j*_ = 0, then ${\mathcal {R}}^{{\mathbf k}} \subset \bigcap _{\{j | k_{j} =0\}} {{\varSigma }}_{j} \subset {{\varSigma }}$. We assume that *α*(*y*) is such that ${\mathcal {R}}^{{\mathbf k}} $ is a submanifold of ${\mathbb {R}}^{d}$ of codimension *m*, where *m* counts the number of elements *k*_*j*_ being equal to zero. For **k** = (*k*_1_,…,*k*_*d*_) we define ${\mathcal {I}}^{{\mathbf k}} = \{ j | k_{j} = 0 \}$, and we let

$$ {\mathcal N}^{{\mathbf k}} = \Bigl\{ {{\boldsymbol\ell}} \in \{ -1, 1 \}^{d} \Big| \ell_{j} \in \{ -1, 1 \} ~\text{if}~k_{j} =0,~ \ell_{j} = k_{j} ~\text{if}~k_{j} \ne0 \Bigr\} $$

which collects the index vectors ***ℓ*** such that ${\mathcal {R}}^{{{\boldsymbol \ell }}}$ touches ${\mathcal {R}}^{{\mathbf k}}$. With this notation we consider the differential-algebraic equation (DAE)

2.2 $$ \begin{array}{@{}rcl@{}} \dot y &=&\sum\limits_{{{\boldsymbol\ell}}\in {\mathcal N}^{{\mathbf k}}} \biggl( \prod\limits_{j\in {\mathcal{I}}^{{\mathbf k}}} \frac{(1+\ell_{j} \lambda_{j} )}2 \biggr) f^{{{\boldsymbol\ell}}} (y)\\ 0 &=& \alpha_{j} (y) , \qquad j\in {\mathcal{I}}^{{\mathbf k}} \end{array} $$

with algebraic variables $\lambda _{j}, j\in {\mathcal {I}}^{{\mathbf k}}$. In the following we denote the right-hand side of the differential equation in (2.2) by *f*^**k**^(*y*,*λ*^**k**^), where *λ*^**k**^ is the vector that collects $\lambda _{j}, j\in {\mathcal {I}}^{{\mathbf k}}$. Differentiating the algebraic constraint of (2.2) with respect to time yields

2.3 $$ 0 = \alpha_{j}^{\prime}(y) f^{{\mathbf k}} (y, \lambda^{{\mathbf k}} ) , \qquad j \in {\mathcal{I}}^{{\mathbf k}} , $$

which represents *m* nonlinear equations in *m* unknowns $\lambda _{j}, j\in {\mathcal {I}}^{{\mathbf k}}$. We assume that the Implicit Function Theorem can be applied to guarantee that locally *λ*^**k**^ can be expressed as function of *y*. This implies that the DAE has index 2. The special case ${\mathcal {I}}^{{\mathbf k}} = \emptyset $ includes classical solutions of (1.3), because in this case ${\mathcal N}^{{\mathbf k}} = \{ {\mathbf {k}} \}$ consists of only one element and the empty product in (2.2) is interpreted as 1.

For *λ*_*j*_ ∈ [− 1, 1] the vector field in (2.2) is a convex combination of the vector fields *f*^***ℓ***^(*y*) (with ${{\boldsymbol \ell }} \in {\mathcal N}^{k}$) which are defined on the open domains touching ${\mathcal {R}}^{{\mathbf k}}$. The solution of (2.2) is therefore a Filippov solution.

#### **Definition 2.1**

Consider an index vector **k** with ${\mathcal {I}}^{{\mathbf k}} \ne \emptyset $ and let $m = | {\mathcal {I}}^{{\mathbf k}} | $ be the cardinality of ${\mathcal {I}}^{{\mathbf k}}$. Then, a solution (*y*,*λ*^**k**^) of the differential-algebraic equation (2.2) is called a codimension-*m* sliding mode in the set ${\mathcal {R}}^{{\mathbf k}}$ as long as *λ*_*j*_ ∈ [− 1, 1] for $j\in {\mathcal {I}}^{{\mathbf k}}$.

For a consistent initial value of (2.2), i.e. $y(0)\in {\mathcal {R}}^{{\mathbf k}}$ and *λ*^**k**^(0) given by (2.3), any technique for the numerical solution of DAE’s of index 2 can be applied. Such techniques are explained in detail in the monographs [2, 19].

#### **Definition 2.2**

A piecewise-smooth, continuous function $y:[0,T]\to {\mathbb {R}}^{n}$ is called a solution of the discontinuous dynamical system (1.3), if there exists a finite partition 0 = *t*_0_ < *t*_1_ < *t*_2_ < … < *t*_*N*_ = *T*, such that the following is true: for every subinterval [*t*_*i*_,*t*_*i*+ 1_] there exists ${\mathbf k}_{i}\in \{ -1, 0,1\}^{d}$ with $m_{i} = | {\mathcal {I}}^{{\mathbf k}_{i}}|$ such that the restriction of *y*(*t*) to this interval is a codimension-*m*_*i*_ sliding mode in the set ${\mathcal {R}}^{{\mathbf k}_{i}}$ (a classical solution if ${\mathcal {I}}^{{\mathbf k}_{i}}= \emptyset $).

### Regularization

**Fig. 1:**
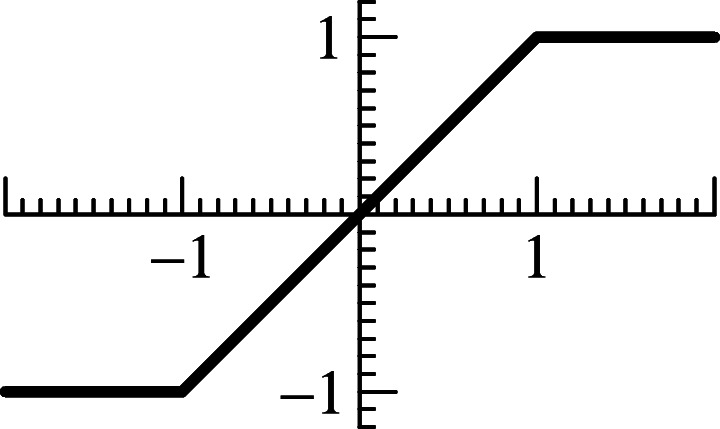
Transition function

We are interested in solutions of (1.3) in the sense of Definition 2.2 that can be considered as the formal limit of a regularized differential equation, where jump discontinuities in the vector field are smoothed out. For this we consider a transition function *π*(*u*), which is assumed to be continuous, piecewise-smooth, and satisfies *π*(*u*) = − 1 for *u* ≤ 1 and *π*(*u*) = 1 for *u* ≥ 1. We also assume that *π*^′^(*u*) > 0 for *u* ∈ (− 1, 1), and that *π*(*u*) is centrally symmetric. A typical example is *π*(*u*) = *u* for |*u*| ≤ 1 (see Fig. 1).

For a discontinuous dynamical system (1.3) we consider the regularization

2.4 $$ \dot y = \sum\limits_{{{\boldsymbol\ell}} \in \{-1, 1\}^{d}} \biggl( \prod\limits_{j=1}^{d} \frac{(1+ \ell_{j}\pi (u_{j}))}2 \biggr) f^{{{\boldsymbol\ell}}} (y) $$

where *u*_*j*_ = *α*_*j*_(*y*)/*ε*. We denote the right-hand side of this regularized differential equation by $f\bigl (y, \pi (u_{1}),{\ldots } , \pi (u_{d} )\bigr )$. The complete phase space (including the discontinuity set *Σ*) is the union of 3^*d*^ sets

2.5 $$ {\mathcal{R}}_{{\varepsilon}}^{{\mathbf k}} = \Big\{ y \in{\mathbb{R}}^{n} \big| |\alpha_{j} (y)| \le {\varepsilon} ~\text{if}~k_{j} =0,~ k_{j}\alpha_{j}(y) >{\varepsilon} ~\text{if}~k_{j} \ne0 \Big\} , $$

where **k** = (*k*_1_,…,*k*_*d*_) with *k*_*j*_ ∈ {− 1, 0,1}. For the case that all *k*_*j*_≠ 0, we have that ${\mathcal {R}}_{{\varepsilon }}^{{\mathbf k}} \subset {\mathcal {R}}^{{\mathbf k}}$, and ***ℓ*** = **k** is the only vector for which the product in (2.4) is non-zero. Therefore, on the set ${\mathcal {R}}_{{\varepsilon }}^{{\mathbf k}}$ the regularization coincides with the differential equation $\dot y = f^{{\mathbf k}} (y)$ of the un-regularized problem.

For **k** with ${\mathcal {I}}^{{\mathbf k}} \ne \emptyset $ the set ${\mathcal {R}}_{{\varepsilon }}^{{\mathbf k}}$ approximates ${\mathcal {R}}^{{\mathbf k}}$. On the set ${\mathcal {R}}_{{\varepsilon }}^{{\mathbf k}}$ only the vectors ${{\boldsymbol \ell }}\in {\mathcal N}^{{\mathbf k}}$ give rise to a non-vanishing product in (2.4). Since *ℓ*_*j*_*π*(*u*_*j*_) = *k*_*j*_*π*(*u*_*j*_) = 1 for $\ell \in {\mathcal N}^{{\mathbf k}}$ and $j\not \in {\mathcal {I}}^{{\mathbf k}}$, the regularized differential (2.4) becomes

2.6 $$ \dot y = \sum\limits_{{{\boldsymbol\ell}} \in {\mathcal N}^{{\mathbf k}}} \biggl( \prod\limits_{j\in {\mathcal{I}}^{{\mathbf k}}} \frac{(1+ \ell_{j}\pi (u_{j}))}2 \biggr) f^{{{\boldsymbol\ell}}} (y)\qquad\text{for}\quad y\in {\mathcal{R}}_{{\varepsilon}}^{{\mathbf k}} , $$

which is in complete analogy to (2.2). If *m* denotes the cardinality of ${\mathcal {I}}^{{\mathbf k}}$, then for *m* = 1 the sum in (2.6) consists of two terms (linear interpolation), for *m* = 2 it consists of four terms (bilinear interpolation), and in general it consists of 2^*m*^ terms.

### Hidden dynamics

A justification of our algorithm is based on the study of the solution of the regularized differential equation, when it is close to an intersection of discontinuity surfaces. In the region ${\mathcal {R}}_{{\varepsilon }}^{{\mathbf k}}$ it follows from (2.6) that *u*_*i*_ = *α*_*i*_(*y*)/*ε* satisfies

2.7 $$ {\varepsilon} \dot u_{i} = \sum\limits_{{{\boldsymbol\ell}} \in {\mathcal N}^{{\mathbf k}}} \biggl( \prod\limits_{j\in {\mathcal{I}}^{{\mathbf k}}} \frac{(1+ \ell_{j}\pi (u_{j}))}2 \biggr) \alpha_{i}^{\prime}(y)f^{{{\boldsymbol\ell}}} (y) ,\qquad i \in {\mathcal{I}}^{\mathbf{k}} , $$

which is a singularly perturbed differential equation. Close to a point $y^{*}\in {\mathcal {R}}^{{\mathbf k}}$ of the discontinuity manifold it can be studied by separating a transient part from the smooth solution. For this we introduce the fast time *τ* = *t*/*ε*, we denote the derivative with respect to *τ* by a prime, and we substitute the constant vector *y*^∗^ for *y*. This yields

2.8 $$ u_{i}^{\prime} = \sum\limits_{{{\boldsymbol\ell}} \in {\mathcal N}^{{\mathbf k}}} \biggl( \prod\limits_{j\in {\mathcal{I}}^{{\mathbf k}}} \frac{(1+ \ell_{j}\pi (u_{j}))}2 \biggr) \alpha_{i}^{\prime}(y^{*})f^{{{\boldsymbol\ell}}} (y^{*}) ,\qquad i \in {\mathcal{I}}^{{\mathbf k}} , $$

which is a regular dynamical system for $u_{i}, i\in {\mathcal {I}}^{{\mathbf {k}}}$. It is called *hidden dynamics* (a term coined in [21]). We expect that this system credibly describes the transient behaviour of the solution of the regularized differential equation.^1^

#### Special case of two intersecting surfaces

We assume that only two components of **k** are zero, say, *k*_1_ = *k*_2_ = 0. We then have ${\mathcal {I}}^{{\mathbf {k}}} = \{ 1, 2 \}$ and ${\mathcal {N}}^{k}$ consists of four elements. The differential (2.8) of the hidden dynamics is then given by, for *i* = 1, 2,

2.9 $$ \begin{array}{@{}rcl@{}} \!\!\!u_{i}^{\prime}& = & \displaystyle \frac 14 \left( \left( 1+ \pi (u_{1})\right)\left( 1+ \pi (u_{2})\right) f_{i}^{1, 1} + \left( 1+ \pi (u_{1})\right)\left( 1- \pi (u_{2})\right) f_{i}^{1, -1}\right.\\ &&\left.+ \left( 1 - \pi (u_{1})\right)\left( 1+ \pi (u_{2})\right) f_{i}^{-1, 1} + \left( 1 - \pi (u_{1})\right)\left( 1 - \pi (u_{2})\right) f_{i}^{-1, -1} \right) , \!\!\! \end{array} $$

where in the notation $f_{i}^{\ell _{1},\ell _{2}} = \alpha _{i}^{\prime } (y^{*}) f^{{{\boldsymbol \ell }}} (y^{*})$ we have omitted the non relevant indices of ***ℓ***. It is of interest for (*u*_1_,*u*_2_) in the unit square [− 1, 1] × [− 1, 1]. We also denote de right-hand side of (2.9) by $g_{i} \bigl (\pi (u_{1}), \pi (u_{2})\bigr )$.

This 2-dimensional system has been discussed in detail in [17, Section 5]: how initial values are determined by the incoming solution, how the behaviour of the solution for $\tau \to \infty $ determines which kind of solution (classical or sliding) will be followed by the regularized equation, how a geometric study of the flow is possible, etc. We note that the right-hand side of (2.9) is a quadratic polynomial in *π*(*u*_1_),*π*(*u*_2_), which vanishes on a hyperbola in the $ \bigl (\pi (u_{1}), \pi (u_{2})\bigr )$-space. Throughout the present work we consider the transition function of Fig. 1.

The study of the hidden dynamics is an essential tool for designing the algorithm proposed in the present paper. A whole monograph [22] is devoted to this topic. Let us also mention the work [23], which concentrates on the 2-dimensional system (2.9). On the basis of singular perturbation theory it discusses stability of sliding, and it shows that there exists at most one stable sliding vector field.

### Scaling invariance

A substitution *α*_*j*_(*y*) → *κ*_*j*_*α*_*j*_(*y*) with *κ*_*j*_ ≥ 1 neither changes the discontinuous hyper-surfaces and the open regions ${\mathcal {R}}^{{\mathbf k}}$ nor the solution of the discontinuous dynamical system (1.3). However, it changes the regularization (2.4) (*u*_*j*_ = *α*_*j*_(*y*)/*ε* is replaced by *κ*_*j*_*u*_*j*_) and therefore also the solution of the regularized differential equation. Consequently, also in the hidden dynamics the expression *π*(*u*_*j*_) is replaced by *π*(*κ*_*j*_*u*_*j*_).

One of our aims is to design an algorithm for the numerical solution of (1.3) that is invariant with respect to such a scaling.

## Solving piecewise-smooth dynamical systems

Typically, a numerical algorithm for solving piecewise-smooth dynamical systems (1.3) is composed of three parts:

- *Computation.* Use any code that permits to solve the index-2 differential-algebraic equation (2.2) starting at consistent initial values. Techniques and codes are well documented in text books like [19] and [2]. In the beginning one is usually concerned with a classical solution, for which ${\mathcal {I}}^{{\mathbf k}} = \emptyset $, so that all $y\in {\mathcal {R}}^{{\mathbf k}}$ are consistent. At a transition point *t*_*i*_ the initial value is determined by continuity.
- *Event location.* The code has to be equipped with an event location algorithm that stops the integration either (i) when the solution enters a new discontinuity surface or (ii) when one of the Lagrange multipliers *λ*_*j*_ leaves the interval [− 1, 1] or (iii) when the solution *λ*^**k**^ of the algebraic system (2.3) ceases to exist in the unit cube or becomes unstable. Since all *λ*_*j*_ are functions of *y*, each of the conditions gives raise to an algebraic relation $g\bigl (y(t) \bigr ) = 0$. Event detection strategies are made for finding such points. This defines a new grid point *t*_*i*_. Algorithms for event location are discussed in [28] (based on the BDF code DASSL) and in [16, 24] (based on the implicit Runge–Kutta code RADAU5).
- *Switching.* As soon as a new transition point *t*_*i*_ is detected, one can check all possible multi-indices **k** which, for the present solution value, give raise to a meaningful solution in the sense of Definition 2.1. In the case of non-uniqueness one can follow all possible solutions (which may be laborious and inefficient) or one can select one of them — but which one? The present article is devoted to a theoretically founded switching criterion based partially on the classification of [17]. It provides a solution that can be considered as the formal limit of a regularized problem.

The present work focuses on the switching algorithm. It is neither our intention to give details on the numerical computation of differential-algebraic equations of index 2 nor to discuss techniques for event location.

## Switching algorithm

The idea is to select a solution that can be considered as the limit solution of a regularized differential equation. For the case that a solution enters a codimension-2 discontinuity the algorithm is based on the classification of [17]. The treatment of the more challenging situation of exiting a codimension-2 discontinuity is new. Depending on whether, on the interval [*t*_*i*− 1_,*t*_*i*_], the solution is a classical solution or a sliding mode, the switching algorithm at *t*_*i*_ is discussed in the following subsections:

- for a *classical solution* in Section 4.1;
- for a *codimension-1 sliding mode* in Section 4.2;
- for a *codimension-2 sliding mode* in Section 4.3.
- for an accumulation of grid points and *spiraling solutions* in Section 4.4.

### Classical solution

**Fig. 2:**
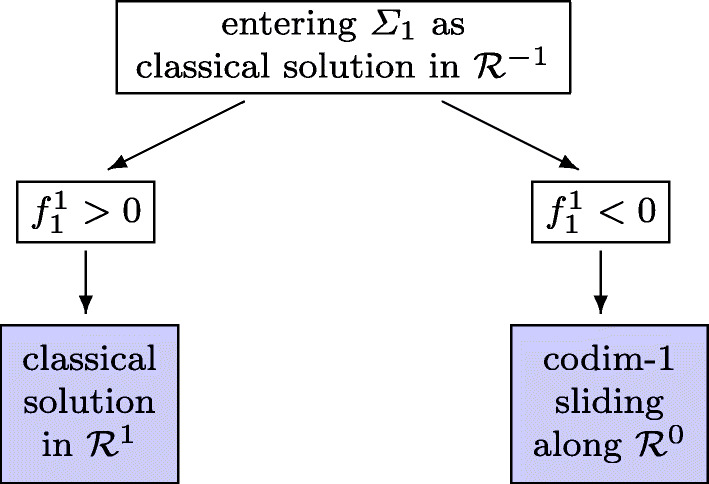
Flowchart of possible switchings from a classical solution

We consider a classical solution of the differential equation (1.3) for *t* ≥ *t*_*i*− 1_ until it enters a discontinuity surface at time *t*_*i*_. Without loss of generality we assume that the discontinuity surface is *Σ*_1_ = {*y*|*α*_1_(*y*) = 0}. Removing irrelevant indices from the vector **k** (for notational convenience), we assume the classical solution to be in ${\mathcal {R}}^{-1} = \{ y | \alpha _{1} (y) < 0 \}$ with vector field *f*^− 1^(*y*). On the opposite side of *Σ*_1_ the vector field is *f*^1^(*y*). We assume that the solution enters transversally the surface *Σ*_1_, so that $f_{1}^{-1}:=\alpha _{1}^{\prime } (y) f^{-1}(y) >0$ at the entry point. We then distinguish the cases, where ${f_{1}^{1}}:=\alpha _{1}^{\prime } (y) f^{1}(y)$ is positive or negative (see Fig. 2). We do not consider the non generic situation, where this expression vanishes.

If ${f_{1}^{1}} > 0$, the only possible solution is classical in the region ${\mathcal {R}}^{1}$. If ${f_{1}^{1}} <0$, there is no classical solution leaving the solution point in *Σ*_1_. The solution in ${\mathcal {R}}^{0} = {{\varSigma }}_{1}$ is defined by the DAE (2.2), i.e.

4.1 $$ \dot y = \frac{(1+\lambda )}{2} f^{1}(y) + \frac{(1-\lambda )}{2} f^{-1}(y) ,\qquad \alpha_{1} (y) =0 . $$

Differentiating the constraint with respect to time yields

$$ \alpha_{1}^{\prime} (y) \Bigl(\frac{(1+\lambda )}{2} f^{1}(y) + \frac{(1-\lambda )}{2} f^{-1}(y)\Bigr) = 0 , $$

which determines *λ* as function of *y*, namely,

4.2 $$ \lambda = \frac{\alpha_{1}^{\prime}(y)f^{-1}(y) + \alpha_{1}^{\prime}(y)f^{1}(y)}{\alpha_{1}^{\prime}(y)f^{-1}(y) - \alpha_{1}^{\prime}(y)f^{1}(y)} . $$

The initial value for (4.1) is defined by continuity for *y*, and for *λ* it satisfies − 1 < *λ* < 1, because $f_{1}^{-1} >0$ and ${f_{1}^{1}} <0$.

### Codimension-1 sliding mode

Suppose that we are concerned with a codimension-1 sliding mode along *Σ*_1_ for *t* ≥ *t*_*i*− 1_. The type of solution can change at some *t*_*i*_ > *t*_*i*− 1_, if either it exits the discontinuity surface *Σ*_1_ or it enters an additional discontinuity surface, say *Σ*_2_. Both situations are discussed in the next two subsections.

#### Exiting *Σ*_1_ from a codimension-1 sliding

**Fig. 3:**
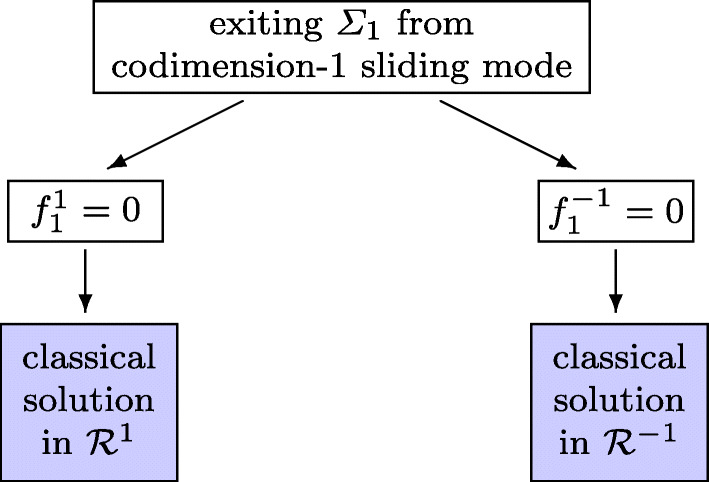
Flowchart of switchings from a codimension-1 sliding mode exiting *Σ*_1_

During the codimension-1 sliding on *Σ*_1_ we have $\alpha _{1}^{\prime }(y) f^{-1}(y) > 0$ and also $\alpha _{1}^{\prime }(y) f^{1}(y) < 0$. This implies that *λ* from (4.2) satisfies *λ* ∈ (− 1, 1). The solution exits this sliding, if *λ* leaves the interval [− 1, 1]. This can happen at *λ* = − 1 (for which $\alpha _{1}^{\prime }(y) f^{-1}(y) $ changes sign from positive to negative) or at *λ* = 1 (for which $\alpha _{1}^{\prime }(y) f^{1}(y) $ changes sign from negative to positive). The solution then continues as classical solution in ${\mathcal {R}}^{-1}$ or ${\mathcal {R}}^{1}$, respectively. The switching is shown in Fig. 3, where we abbreviate the expressions $\alpha _{1}^{\prime }(y) f^{-1}(y) $ and $\alpha _{1}^{\prime }(y) f^{1}(y) $ at the exit point *y* by $f_{1}^{-1}$ and ${f_{1}^{1}}$.

#### Entering the intersection *Σ*_1_ ∩*Σ*_2_

As before we disregard irrelevant indices from the index vector **k**, and we keep only those corresponding to *Σ*_1_ and *Σ*_2_. We consider a codimension-1 sliding along ${\mathcal {R}}^{0,-1} = \{ y | \alpha _{1} (y)=0, \alpha _{2} (y) < 0 \}$, and we generically assume that

**Fig. 4:**
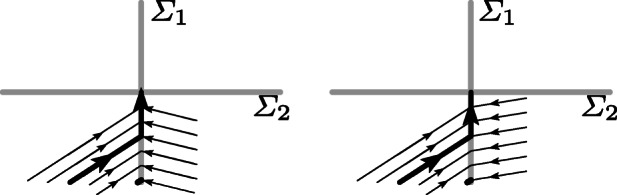
Entering the codimension-2 manifold

4.3 $$ \begin{array}{@{}rcl@{}} &&f_{1}^{-1, -1} > 0, \qquad f_{1}^{1, -1} <0, \qquad f_{2}^{-1, -1} >0, \\ &&f_{1}^{-1, -1} f_{2}^{1, -1} - f_{1}^{1, -1} f_{2}^{-1, -1} > 0 , \end{array} $$

where we use the notation $f_{j}^{{\mathbf k}} = \alpha _{j}^{\prime }(y) f^{{\mathbf k}} (y)$ for *j* ∈ {1, 2} and **k** = (*k*_1_,*k*_2_) (all vector fields are evaluated at the entry point). The first two inequalities of (4.3) mean that both vector fields, *f*^− 1, − 1^ and *f*^1, − 1^, point towards ${\mathcal {R}}^{0,-1}$. To have a sliding motion along ${\mathcal {R}}^{0,-1}$ in direction of the intersection *Σ*_1_ ∩*Σ*_2_ at least one among $f_{2}^{-1, -1}$ and $f_{2}^{1, -1}$ has to be positive. Without loss of generality we assume $f_{2}^{-1, -1}>0$. Figure 4 illustrates the situations $f_{2}^{1, -1}>0$ (left picture) and $f_{2}^{1, -1}<0$ (right picture). In the second case the last inequality of (4.3) guarantees that the sliding vector field points upwards.

**Fig. 5:**
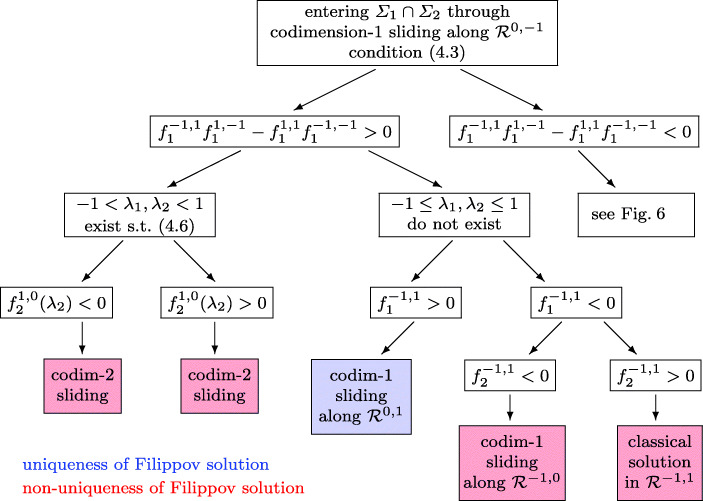
Flowchart of switchings from a codimension-1 entering *Σ*_1_ ∩*Σ*_2_. In the case of multiple solutions of (4.6), *λ*_2_ is the value that is closest to − 1. Here, and in the following, the term “Filippov solution” means a solution according to Definition 2.2

**Fig. 6:**
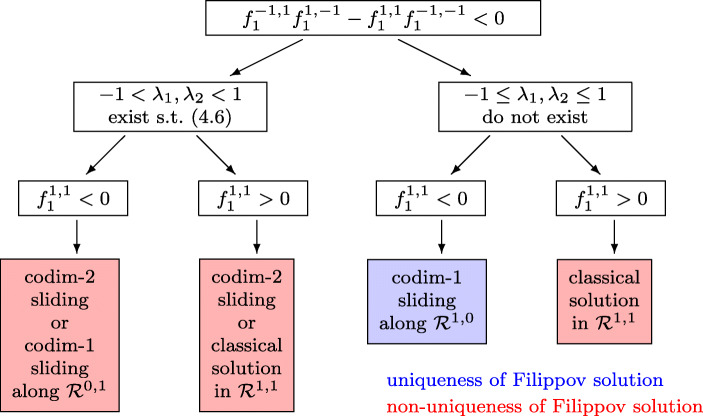
Flowchart of switchings from a codimension-1 entering *Σ*_1_ ∩*Σ*_2_ (cont.). Condition (4.4) is assumed in addition to the assumption (4.3) of the flowchart of Fig. 5

The switching algorithm of Fig. 5 is based on [17, Theorem 6.1], that of Fig. 6, which completes Fig. 5, is based on [17, Theorem 6.2] (see Section 5.1 for more details). The algorithm of Fig. 6, is valid under the additional condition

4.4 $$ f_{2}^{1, -1} < 0 , $$

which can be assumed without loss of generality, because the case $f_{2}^{1, -1} > 0 $ can be reduced to that of Fig. 5 by symmetry considerations.

The algorithm presented in the two figures needs some more explanations. In addition to classical and codimension-1 solutions we have to consider codimension-2 solutions. They are defined by

4.5 $$ \begin{array}{@{}rcl@{}} \dot y &=& \frac 14 \left( (1+\lambda_{1} )(1+\lambda_{2} ) f^{1, 1}(y) + (1+\lambda_{1} )(1-\lambda_{2} ) f^{1, -1}(y)\right.\\ &&\left.+(1-\lambda_{1} )(1+\lambda_{2} ) f^{-1, 1}(y) + (1-\lambda_{1} )(1-\lambda_{2} ) f^{-1, -1}(y)\right) \end{array} $$

subject to the algebraic constraints *α*_1_(*y*) = 0 and *α*_2_(*y*) = 0. The right-hand side of (4.5) is denoted by *f*^0,0^(*y*,*λ*_1_,*λ*_2_). Differentiating the algebraic relations with respect to time yields (when multiplied by 4)

4.6 $$ \begin{array}{@{}rcl@{}} && \alpha_{j}^{\prime} (y) \left( (1+\lambda_{1} )(1+\lambda_{2} ) f^{1, 1}(y) + (1+\lambda_{1} )(1-\lambda_{2} ) f^{1, -1}(y)\right.\\ &&\left.\quad +(1-\lambda_{1} )(1 + \lambda_{2} ) f^{-1, 1}(y) + (1 - \lambda_{1} )(1 - \lambda_{2} ) f^{-1, -1}(y)\right) = 0 \end{array} $$

for *j* ∈ {1, 2}. We also use the notation *g*_*j*_(*y*,*λ*_1_,*λ*_2_) = 0 for this equation. For the existence of a locally unique solution (*λ*_1_,*λ*_2_) of the system (4.6), we assume that the Implicit Function Theorem can be applied, which means that the 2 × 2 matrix

4.7 $$ G(y,\lambda_{1},\lambda_{2}) = \Bigl(\alpha_{j}^{\prime}(y) \frac{\partial}{\partial \lambda_{p}} f^{0,0}(y,\lambda_{1},\lambda_{2}) \Bigr)_{j,p=1}^{2} $$

is invertible. For a fixed value of *y* the equation (4.6) represents a hyperbola with vertical and horizontal asymptotes in the (*λ*_1_,*λ*_2_)-space. We are only interested in values (*λ*_1_,*λ*_2_) lying in the square [− 1, 1] × [− 1, 1] (which we sometimes call unit square).

When, in the algorithms of Figs. 5 and 6, we write that a solution (*λ*_1_,*λ*_2_) of (4.6) exists (or not) in the unit square, we mean only solutions on the branch of the first hyperbola (*j* = 1) that crosses the bottom side of the square. The expression $f_{2}^{1, 0} (\lambda _{2})$, appearing in the switching algorithm, is defined by $f_{2}^{1, 0} (\lambda _{2}) = \alpha _{2}^{\prime }(y) f^{1, 0} (y,\lambda _{2})$, and *f*^1, 0^(*y*,*λ*_2_) is the vector field of (2.2) for **k** = (1, 0).

##### *Remark 4.1*

In the situation, where the algorithm of Figs. 5 and 6 proposes a codimension-2 sliding, the discussion of [17] shows that in the beginning of the sliding the solution (*λ*_1_,*λ*_2_) of (4.6) is such that the determinant of *G*(*y*,*λ*_1_,*λ*_2_) is positive and at least one of its diagonal elements is negative. This is important for the strategy in Section 4.3.3.

### Codimension-2 sliding mode

Suppose that there is a codimension-2 sliding mode along *Σ*_1_ ∩*Σ*_2_ for *t* ≥ *t*_*i*− 1_. It is characterized by the existence of $\bigl (\lambda _{1} (t), \lambda _{2} (t)\bigr ) \in (-1, 1)^{2}$ satisfying the polynomial system (4.6). The type of solution can change at some *t*_*i*_ > *t*_*i*− 1_, if either (a) the pair $\bigl (\lambda _{1} (t), \lambda _{2} (t)\bigr )$ leaves the unit square (− 1, 1)^2^, (b) the solution $\bigl (\lambda _{1} (t), \lambda _{2} (t)\bigr )$ of (4.6) becomes double and ceases to exist, (c) the matrix (4.7) changes stability (see Remark 4.1), (d) the sliding mode enters an additional discontinuity surface, say *Σ*_3_. The algorithms for the situations (a), (b), and (c) are presented in the following subsections, their justification is discussed in Section 5. In this paper we do not consider the situation (d), because we are not aware of results on the limit solution of the regularized differential equation close to a codimension-3 discontinuity surface.

#### Exiting *Σ*_1_ ∩*Σ*_2_ from a codimension-2 sliding — type (a)

We assume that at *t* = *t*_*i*_ the pair $\bigl (\lambda _{1}(t), \lambda _{2}(t)\bigr )$ of the system (4.6) leaves the unit square at one side (generically, we can exclude the corners). Without loss of generality we can assume that

**Fig. 7:**
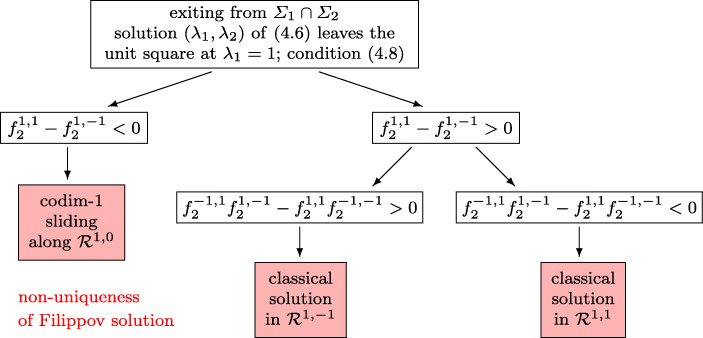
Flowchart for exiting a codimension-2 sliding of type (a); c.f., case (A) of [18]

4.8 $$ \lambda_{1} (t_{i}) = 1, \qquad \dot \lambda_{1} (t_{i}) > 0, \qquad -1 < \lambda_{2} (t_{i}) < 1 $$

hold. The proposed switching, based on [18, Theorems 2 and 3], is shown in Fig. 7. All vector fields are evaluated at the exit point. According to the decision tree the solution of the discontinuous problem either continues, beyond the exit point, as a codimension-1 sliding mode or as a classical solution.

Note that all situations of Fig. 7 admit further solutions (classical or codimension-1). The proposed algorithm chooses the solution that can be realized as the limit of a regularized differential equation.

#### Exiting *Σ*_1_ ∩*Σ*_2_ from a codimension-2 sliding — type (b)

We assume that the two (real) solutions of the system (4.6) coalesce in the unit square and disappear at *t* = *t*_*i*_. This implies that at this time instant the two hyperbolas in the (*λ*_1_,*λ*_2_)-space are tangential at $\bigl (\lambda _{1}(t_{i}),\lambda _{2}(t_{i})\bigr )$. Without loss of generality we assume that the hyperbolas have positive slope which, expressed in terms of the vector fields, is (see Lemma 6.3 of [17])

4.9 $$ f_{1}^{-1, 1}f_{1}^{1, -1} - f_{1}^{1, 1}f_{1}^{-1, -1} < 0, \qquad f_{2}^{-1, 1}f_{2}^{1, -1} - f_{2}^{1, 1}f_{2}^{-1, -1} < 0 $$

(otherwise we reflect the picture at the vertical axis, i.e. change the sign of *λ*_1_). Moreover, we assume that

**Fig. 8:**
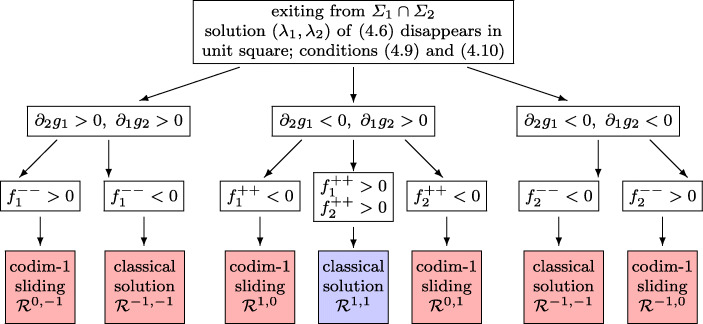
Flowchart for exiting a codimension-2 sliding of type (b); for brevity we use the notation $f_{i}^{++}$ and $f_{i}^{--}$ for $f_{i}^{1, 1}$ and $f_{i}^{-1, -1}$, respectively. All functions are evaluated at the collapsing stationary point

4.10 $$ \text{ the hyperbola corresponding to $\alpha_{2}(y)$ lies above that of $\alpha_{1} (y)$} $$

(otherwise we exchange *α*_1_ and *α*_2_). Denoting the left-hand expression in (4.6) by *g*_*j*_(*λ*_1_,*λ*_2_) and the derivative with respect to *λ*_*i*_ by *∂*_*i*_, we distinguish cases according to the signs of *∂*_*i*_*g*_*j*_. Figure 8 gives a complete characterization of all possible situations (we shall explain later in Section 5.2 that the apparantly missing situation *∂*_2_*g*_1_ > 0,*∂*_1_*g*_2_ < 0 cannot arise at a vanishing stationary point). As in previous figures the boxes in red indicate that more than one Filippov solutions are possible. There is only one situation (blue box) with a unique solution. In the case of non-uniqueness our algorithm selects the solution which can be interpreted as the formal limit of the solution of a regularized problem.

#### Exiting *Σ*_1_ ∩*Σ*_2_ from a codimension-2 sliding — type (c)

A stationary point of the hidden dynamics (2.9) (corresponding to a solution (*λ*_1_,*λ*_2_) of (4.6)) is asymptotically stable if both eigenvalues of (4.7) have negative real part. This is equivalent to

4.11 $$ \det G (y,\lambda_{1},\lambda_{2}) > 0\qquad\text{and}\qquad \text{trace } G (y,\lambda_{1},\lambda_{2}) < 0 . $$

As explained in [17, Section 8] the trace of the matrix *G* is not scaling invariant. It is shown that, if at least one of the diagonal elements of *G* is negative, there exists a scaling that makes the stationary point asymptotically stable. The condition for a stationary point to be asymptotically stable after a suitable scaling of the constraints therefore becomes

4.12 $$ \det G (y,\lambda_{1},\lambda_{2}) > 0\qquad\text{and}\qquad \min_{i=1, 2} G_{i,i}(y,\lambda_{1},\lambda_{2}) < 0 , $$

where *G*_*i*,*j*_ stands for the elements of the matrix *G* of (4.7); see also Remark 4.1. Our (scaling invariant) strategy is to exit a codimension-2 sliding, if one of the two conditions in (4.12) becomes violated.

1. Assume first that at time *t* = *t*_*i*_ we have $\det G (y^{*},\lambda _{1}^{*},\lambda _{2}^{*}) = 0$ (i.e. $\det G (y,\lambda _{1},\lambda _{2}) $ changes from positive to negative), while the second condition of (4.12) still holds. Due to the special structure of (4.6) both of its solutions coalesce at *t* = *t*_*i*_ and, while $\det G (y,\lambda _{1},\lambda _{2}) $ changes from positive to negative for the actual solution, it changes from negative to positive for the other solution. In this situation, we propose to continue with a codimension-2 sliding, and we take for (*λ*_1_,*λ*_2_) the solution of (4.6) for which the determinant of *G*(*y*,*λ*_1_,*λ*_2_) is positive.

(2) Assume next that the second condition of (4.12) is violated, but we still have $\det G (y,\lambda _{1},\lambda _{2}) > 0$. Generically, one among the diagonal elements of *G* is then positive and the other equals zero. Without loss of generality we assume that at the transition point we have $G_{1, 1}(y^{*},\lambda _{1}^{*},\lambda _{2}^{*}) =0$ and $G_{2,2}(y^{*},\lambda _{1}^{*},\lambda _{2}^{*}) >0$. The condition $G_{1, 1}(y^{*},\lambda _{1}^{*},\lambda _{2}^{*}) =0$ is only possible, if the hyperbola *g*_1_(*y*^∗^,*λ*_1_,*λ*_2_) = 0 degenerates (i.e. it is the union of the horizontal asymptote $\lambda _{2} = \lambda _{2}^{*}$ and of the vertical asymptote). By changing the sign of *α*_1_(*y*) and/or of *α*_2_(*y*) we can assume that

4.13 $$ f_{1}^{-1, -1}<0,\qquad f_{1}^{-1, 1} >0,\qquad G_{1, 2}(y^{*},\lambda_{1}^{*},\lambda_{2}^{*})>0 . $$

**Fig. 9:**
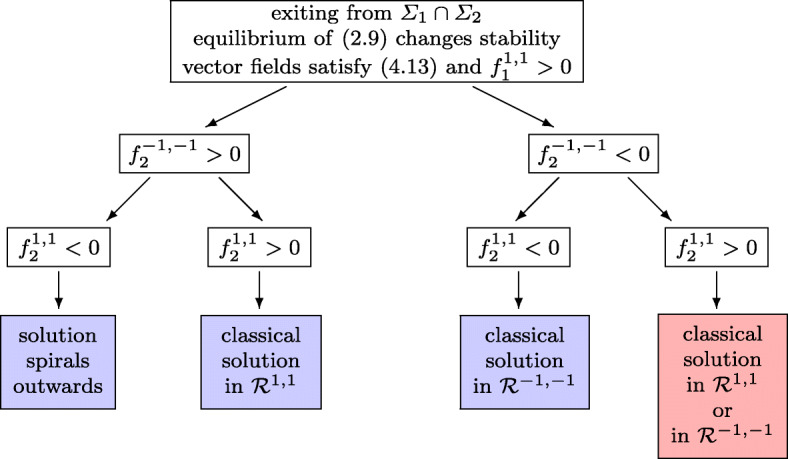
Flowchart of possible exits from a codimension-2 sliding under the assumption that $\partial _{1} g_{1}(y^{*},\lambda _{1}^{*},\lambda _{2}^{*})=0$ and $\partial _{2} g_{2}(y^{*},\lambda _{1}^{*},\lambda _{2}^{*})>0$. The vertical asymptote of *g*_1_ = 0 is outside the unit square

**Fig. 10:**
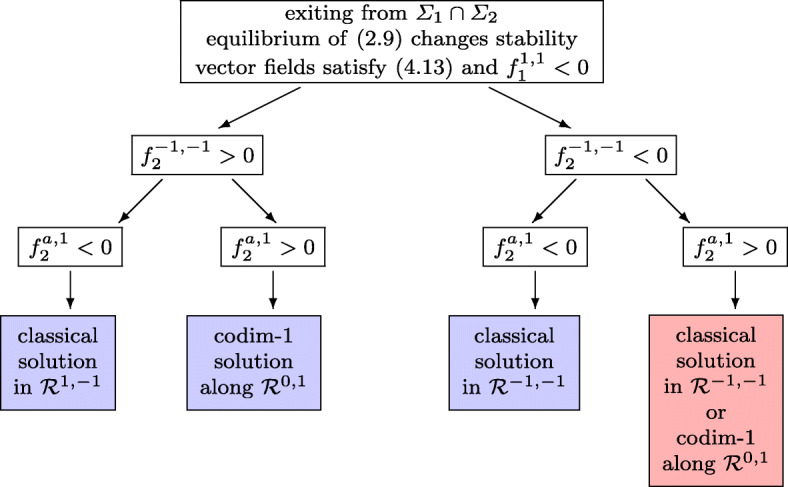
Flowchart of possible exits from a codimension-2 sliding under the assumption that $\partial _{1} g_{1}(y^{*},\lambda _{1}^{*},\lambda _{2}^{*})=0$ and $\partial _{2} g_{2}(y^{*},\lambda _{1}^{*},\lambda _{2}^{*})>0$. The vertical asymptote of *g*_1_ = 0 is inside the unit square to the right of $\lambda _{1}^{*}$

This means that for $-1\le \lambda _{1} \le \lambda _{1}^{*}$ the function *g*_1_(*y*^∗^,*λ*_1_,*λ*_2_) takes positive values above the horizontal asymptote and has the vertical asymptote outside the interval $[-1, \lambda _{1}^{*}]$. We now distinguish between two situations according to the sign of $f_{1}^{1, 1}$. For $f_{1}^{1, 1}>0$ the vertical asymptote of *g*_1_(*y*^∗^,*λ*_1_,*λ*_2_) = 0 is outside the unit square, and for $f_{1}^{1, 1}< 0$ it lies between $\lambda _{1}^{*}$ and + 1. The type of solutions beyond the switching point *t*_*i*_ are shown in Fig. 9 for the case $f_{1}^{1, 1}>0$, and in Fig. 10 for the case $f_{1}^{1, 1}<0$. In this figure we use the abbreviation $f_{2}^{a ,1}= g_{2} ({\lambda _{1}^{a}} ,1)$, where ${\lambda _{1}^{a}}$ denotes the abscissa of the vertical asymptote corresponding to *g*_1_(*y*^∗^,*λ*_1_,*λ*_2_) = 0.

### Accumulation of grid points, entering *Σ*_1_ ∩*Σ*_2_ through spiraling

We consider the situation, where a solution of (1.3) enters the intersection at *y* ∈*Σ*_1_ ∩*Σ*_2_ by spiraling inwards. This can be clockwise or counterclockwise. Assuming the second, this is the case, if the vector fields, evaluated at *y*, satisfy

4.14 $$ \begin{array}{c} f_{1}^{-1, -1} >0,\quad f_{1}^{1, -1} >0 ,\quad f_{1}^{1, 1} <0, \quad f_{1}^{-1, 1} <0 \\ f_{2}^{-1, -1} <0,\quad f_{2}^{1, -1} >0 ,\quad f_{2}^{1, 1} >0, \quad f_{2}^{-1, 1} <0 , \end{array} $$

and if the contractivity condition

4.15 $$ 0< \gamma < 1 \quad\text{with}\quad \gamma = \frac{f_{2}^{-1, -1}}{f_{1}^{-1, -1}}\cdot \frac{f_{1}^{1, -1}}{f_{2}^{1, -1}}\cdot \frac{f_{2}^{1, 1}}{f_{1}^{1, 1}} \cdot \frac{f_{1}^{-1, 1}}{f_{2}^{-1, 1}} $$

holds. Under these two assumptions the solution of the discontinuous system (1.3) converges to *Σ*_1_ ∩*Σ*_2_ in finite time. It spirals around *Σ*_1_ ∩*Σ*_2_ and produces an infinity of grid points that converge geometrically to the entry point. From there on we have a codimension-2 sliding.

## Justification of the algorithm

In the situation of Sections 4.1, 4.2.1, and 4.4, we have uniqueness of the solution (classical and sliding modes) beyond the new grid point *t*_*i*_, and nothing has to be justified for the algorithm. This is not necessarily the case for the situation of Sections 4.2.2 and 4.3, where the solution enters or exits a codimension-2 hyper-surface.

The philosophy of the presented algorithm is that in the situation of non-uniqueness we choose a solution that can be interpreted as the limit for *ε* → 0 of the solution of a regularization. The combined system (2.6)–(2.7) is a singularly perturbed differential equation which is typically studied by asymptotic expansions in powers of *ε* and by separating slow and fast dynamics. This, however, is not always possible in the present context. The experiment of [18, Section 4.2] even demonstrates the lack of an expansion in integer powers of *ε*.

On the other hand, close to a value *y*^∗^ in the discontinuity manifold, it is expected that the hidden dynamics (2.8) reproduces well the bahaviour of the solution of the discontinuous equation. For the case that *α*_*j*_(*y*) is an affine function of *y* and that the vector fields are constant in a neighbourhood of *y*^∗^, the solution of (2.8) describes the functions *u*_*i*_ = *α*_*i*_(*y*)/*ε* without any error.

We thus trust the hidden dynamics and we propose to select the solution after a switching point according to the behaviour the hidden dynamics. This implies that we have a transition to (c.f. [17, Section 5.2])

- a *classical solution*, if both solution components of (2.9) are unbounded,
- a *codimension-1 sliding*, if one solution component is unbounded and the other converges for $\tau \to \infty $ to a value in (− 1, 1),
- a *codimension-2 sliding*, if the pair $\bigl (u_{1}(\tau ),u_{2}(\tau )\bigr )$ converges to a point in the unit square.

### Justification of the algorithms of Sections 4.2.2 and 4.3.1

The algorithms of Figs. 5 and 6 are just a transcription of Theorems 6.1 and 6.2 of [17], where the conditions are written in terms of the four vector fields rather than in terms of the vector field of the hidden dynamics.

The assumption (4.3) is equivalent to (6.1) of [17]. By Lemma 6.3 of [17] the condition *∂*_2_*g*_1_(*u*_1, 0_,− 1) < 0 (left turning situation) in [17] is equivalent to $f_{1}^{-1, 1}f_{1}^{1, -1} - f_{1}^{1, 1}f_{1}^{-1, -1} >0$ (top left formula in Fig. 5). Item (a) of Theorem 6.1 in [17] corresponds to the existence of a solution of (4.6) in the unit square. In the notation of the present work the expression *g*_*β*_(1, *u*^∗^) of [17, Theorem 6.1] is equal to $g_{2} (1, \lambda _{2}) = f_{2}^{1, 0}(\lambda _{2} )$, which appears in Fig. 5. The situation in [17, Theorem 6.1], where the solution of the hidden dynamics approaches a limit cycle around a stationary point, corresponds to high oscillations of amplitude ${\mathcal O} ({\varepsilon } )$ in the solution of the regularized differential equation, and to a codimension-2 sliding in the discontinuous system. Items (b) and (c) of Theorem 6.1 in [17] correspond to the part in Fig. 6, where the system (4.6) does not have a solution in the unit square that lies on the branch of the hyperbola crossing the bottom line of the square.

The algorithm of Fig. 7 is a transcription of the statements in Theorems 2 and 3 of [18]. We note that the expressions $\beta ^{\prime }(y_{0}^{*}) \partial _{u} f(y_{0}^{*},1, v_{0}^{*})$ and $\beta ^{\prime }(y_{0}^{*}) \partial _{v} f(y_{0}^{*},1, v_{0}^{*})$ correspond to *∂*_1_*g*_2_(1, *λ*_2_) and *∂*_2_*g*_2_(1, *λ*_2_) in the notation of the present work. Since *g*_2_(*λ*_1_,*λ*_2_) is an affine function in each of its variables, the sign of *∂*_2_*g*_2_(1, *λ*_2_) is the same as that of $f_{1}^{1, 1}-f_{2}^{1, -1}$, and the sign of *∂*_1_*g*_2_(1, *λ*_2_) is the same as that of $f_{2}^{-1, 1}f_{2}^{1, -1} - f_{2}^{1, 1}f_{2}^{-1, -1}$.

### Justification of the algorithm of Section 4.3.2

**Fig. 11:**
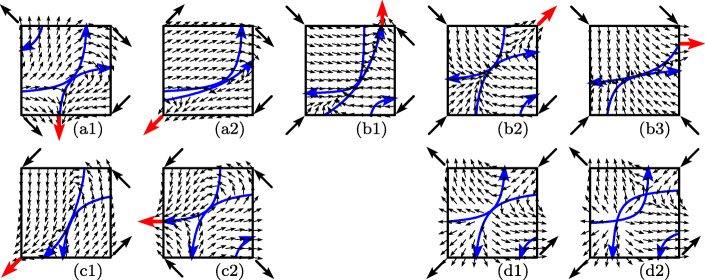
Vector field of the hidden dynamics in the situation where both stationary points coalesce. The first seven pictures correspond to the colored boxes of Fig. 8 in the same order. Red arrows represent the solution after the switching

The first seven pictures of Fig. 11 show the vector fields corresponding to the seven situations of Fig. 8. The hyperbolas *g*_1_ = 0 and *g*_2_ = 0 are drawn in blue. By (4.9) both hyperbolas have positive slope and by (4.10) the hyperbola for *g*_2_ = 0 lies above that for *g*_1_ = 0. We mark the hyperbolas with an arrow which make them to an oriented path. To the left of the hyperbola *g*_1_ = 0 we have by convention *g*_1_ > 0 so that the vector field points to the right, and on the other side the vector field points to the left. Similarly, to the left of *g*_2_ = 0 the vector field points upwards, and downwards on the other side.

We start with the assumption *∂*_2_*g*_1_ > 0, *∂*_1_*g*_2_ > 0. The branches of the hyperbolas passing through the vanishing stationary point are directed upwards. This can be observed in the pictures (a1) and (a2). In the picture (a1), where $f_{1}^{--} >0$, the solution starting at the vanishing stationary point leaves the unit square at the bottom side. This gives rise to a codimension-1 sliding in ${\mathcal {R}}^{0,-1}$. In this situation (again picture (a1)) there is also a classical solution leaving the intersection into the region ${\mathcal {R}}^{-1, 1}$, which however cannot be realized as the limit of a regularization. If $f_{1}^{--} <0$ (picture (a2)), the solution leaves the unit square at the lower left corner, which gives rise to a classical solution in ${\mathcal {R}}^{-1, -1}$. If the branch of the hyperbola *g*_2_ = 0 would leave the unit square at the right side, there would be another classical solution in ${\mathcal {R}}^{1, 1}$, which however is irrelevant.

All other situations, namely (b1), (b2), (b3), (c1), (c2), can be explained similarly by looking at the vector fields in Fig. 11. All of them, with the exception of (b2), admit a second (non relevant) solution by slightly modifying the hyperbolas. For example, in the situation (b1) one can change the hyperbola *g*_1_ = 0 such that it enters at the left side and such that its second branch surrounds the lower right corner. Consequently, there is also a classical solution in ${\mathcal {R}}^{1, -1}$. Slight modifications of the hyperbolas permit to produce a classical solution in ${\mathcal {R}}^{-1, 1}$ for (b3), a classical solution in ${\mathcal {R}}^{1, 1}$ for (c1), and a classical solution in ${\mathcal {R}}^{1, -1}$ for (c2).

The last two pictures of Fig. 11 treat the situation *∂*_2_*g*_1_ > 0, *∂*_1_*g*_2_ < 0. The picture (d1) gives the impression that more than one solution, starting at the vanishing stationary point, are possible. However, when looking at the situation just before the stationary points vanish (picture (d2)), one sees that both stationary points are unstable and that there is no bounded limit cycle in the unit square. Therefore, this situation cannot occur at the end of a codimension-2 sliding.

### Justification of the algorithm of Section 4.3.3

In the situation of the first three pictures of Figs. 9 and 10 there is exactly one solution exiting the codimension-2 hyper-surface. Let us nevertheless briefly discuss the hidden dynamics in these situations. The condition $G_{1, 1}(y^{*},\lambda _{1}^{*},\lambda _{2}^{*})=0$, which in terms of (2.9) reads $\partial _{1} g_{1} (u_{1}^{*},u_{2}^{*}) =0$, implies that the hyperbola *g*_1_(*u*_1_,*u*_2_) = 0 is degenerate. It is the union of the horizontal and vertical asymptote. The assumption (4.13) implies that at the left side of the unit square and in a neighbourhood of the stationary point $(u_{1}^{*},u_{2}^{*})$ the hyperbola *g*_1_(*u*_1_,*u*_2_) = 0 is oriented to the right. The positivity of $\det G$ and of *G*_2,2_ imply that the hyperbola *g*_2_(*u*_1_,*u*_2_) = 0 crosses the stationary point $(u_{1}^{*},u_{2}^{*})$ from bottom left to top right.

**Fig. 12:**
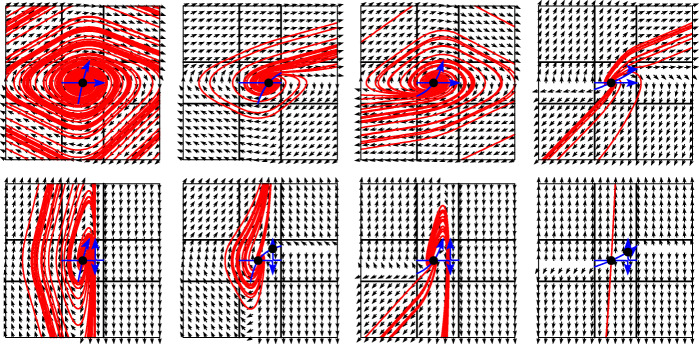
Vector field of the hidden dynamics for the situations discussed in Fig. 9 (upper pictures) and in Fig. 10 (lower pictures). Twenty solutions corresponding to initial values that are random perturbations of the equilibrium $(u_{1}^{*},u_{2}^{*} )$ are plotted in red

The additional assumption $f_{1}^{1, 1}>0$ in Fig. 9 implies that the vertical asymptote of *g*_1_(*u*_1_,*u*_2_) = 0 is outside the unit square. The upper pictures of Fig. 12 illustrate the four situations of Fig. 9. The vector field of the hidden dynamics is shown on the unit square and on a neighbourhood of it. The oriented hyperbolas are indicated in blue, and 20 solutions with randomly chosen initial values 10^− 6^-close to the stationary point are plotted in red. An inspection of the vector field shows that the solutions spiral outwards in the first picture. Apparently, there is an infinity of solutions starting at *t* = *t*_*i*_ at the point *y*^∗^. In the second and third situations the solutions all tend to a classical solution in ${\mathcal {R}}^{1, 1}$ and ${\mathcal {R}}^{-1, -1}$, respectively.

**Fig. 13:**
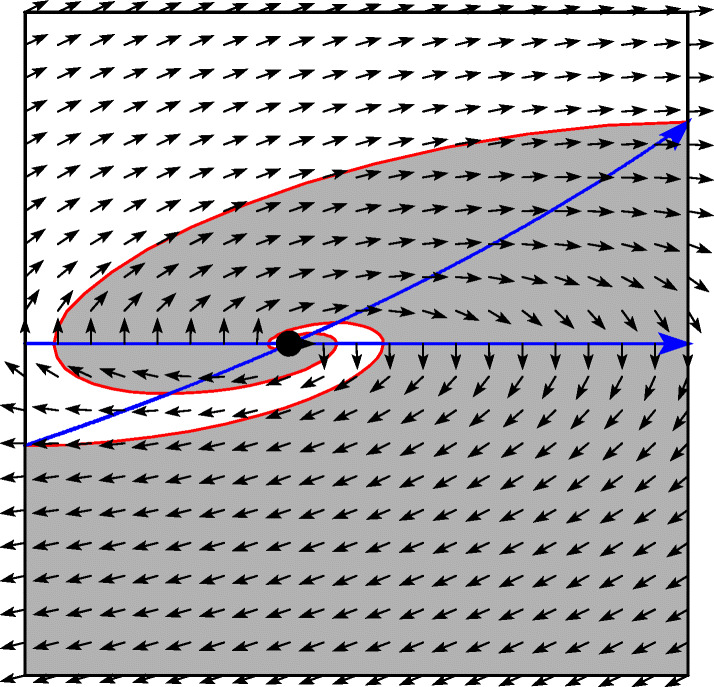
Basin of attraction for the two classical solutions

The fourth picture indicates the existence of two classical solutions, one in ${\mathcal {R}}^{1, 1}$ and the other in ${\mathcal {R}}^{-1, -1}$. Figure 13 shows the basin of attraction of the two solutions. For any initial value in the grey region the solution converges to the classical solution in ${\mathcal {R}}^{-1, -1}$, and for initial values in the white region it converges to ${\mathcal {R}}^{1, 1}$. This clearly shows that there is non-uniqueness of the solution of the discontinuous problem.

The assumption $f_{1}^{1, 1}<0$ in Fig. 10 implies that the vertical asymptote of *g*_1_(*u*_1_,*u*_2_) = 0 is inside the unit square. The lower pictures of Fig. 12 illustrate the four situations of Fig. 10. The second, third, and fourth pictures are similar as before with the exception that the classical solution in ${\mathcal {R}}^{1, 1}$ is now a codimension-1 sliding along ${\mathcal {R}}^{0,1}$. This is because the solution of the hidden dynamics cannot cross the vertical asymptote.

An explanation for the first picture of the lower row in Fig. 12 is more tricky. The solution spirals outwards around the stationary point, but remains to the left of the asymptote. On the other hand, the only stable solution leaving the square is classical in the region ${\mathcal {R}}^{1, -1}$, which is to the right of the asymptote. How can we reach this solution? The reason is that *G*_1, 1_(*y*,*λ*_1_,*λ*_2_) is negative before *t* = *t*_*i*_, but positive after it. Hence, immediately after *t* = *t*_*i*_ the hyperbola *g*_1_(*u*_1_,*u*_2_) = 0 is not degenerate and the vertical asymptote is no longer a separation of solutions of the hidden dynamics. After a few spirals around the stationary point, the solution can escape to the right and follow the classical solution in ${\mathcal {R}}^{1, -1}$.

## Some details for an implementation

Every code for solving ordinary differential equations having an option for event location is suitable for using the algorithm of the present work.

*Solving the algebraic system.* The most convenient way of computing the sliding modes is to solve the algebraic system (2.3) with respect to the *λ*_*j*_, and to insert the result into (2.2), which then gives an ordinary differential equation. For codimension-1 an explicit formula for *λ*_1_ is given by (4.2). For codimension-2 the system (4.6) presents two quadratic equations for *λ*_1_ and *λ*_2_. A suitable linear combination permits to eliminate the product *λ*_1_*λ*_2_ and gives a linear relation between *λ*_1_ and *λ*_2_. Inserted into the original equation this yields a quadratic equation in one variable. We thus get two solutions of the system. One has to choose the solution for which the determinant of the matrix (4.7) is positive.

### Spiraling solutions

When the solution spirals inwards to (or outwards from) a codimension-2 discontinuity hyper-surface we are concerned with an infinity of accumulating transition points. In practice this can be treated as follows (see [17, Section 7]).

Assume that we have detected a point *y*_0_ for which *α*_2_(*y*_0_) = 0 and *α*_1_(*y*_0_) = −*δ* with 0 < *δ* ≪ 1 very small. Assume further that, close to *Σ*_1_ ∩*Σ*_2_, the four vector fields are constant and satisfy (4.14) and (4.15). After a lap around *Σ*_1_ ∩*Σ*_2_ the solution is, up to first order in *δ*,

6.1 $$ y_{1} = y_{0} + {t_{1}^{0}} f^{-1, -1} + {t_{2}^{0}} f^{1, -1} + {t_{3}^{0}} f^{1, 1} + {t_{4}^{0}} f^{-1, 1} $$

where

$$ \begin{array}{lll} {y_{0}^{1}} = y_{0} + {t_{1}^{0}} f^{-1, -1}, & {t_{1}^{0}} = \delta / f_{1}^{-1, -1}, & \alpha_{1}({y_{0}^{1}})=0,\quad \alpha_{2} ({y_{0}^{1}}) = {t_{1}^{0}} f_{2}^{-1, -1}\\ {y_{0}^{2}} = {y_{0}^{1}} + {t_{2}^{0}} f^{1, -1}, & {t_{2}^{0}} =- {t_{1}^{0}} f_{2}^{-1, -1} / f_{2}^{1, -1}, & \alpha_{1}({y_{0}^{2}})={t_{2}^{0}} f_{1}^{1, -1},\quad \alpha_{2} ({y_{0}^{2}}) = 0\\ {y_{0}^{3}} = {y_{0}^{2}} + {t_{3}^{0}} f^{1, 1}, & {t_{3}^{0}} = -{t_{2}^{0}} f_{1}^{1, -1} / f_{1}^{1, 1}, & \alpha_{1}({y_{0}^{3}})=0,\quad \alpha_{2} ({y_{0}^{3}}) = {t_{3}^{0}} f_{2}^{-1, -1}\\ {y_{0}^{4}} = {y_{0}^{3}} + {t_{4}^{0}} f^{-1, 1}, & {t_{4}^{0}} = -{t_{3}^{0}} f_{2}^{1, 1} / f_{2}^{-1, 1}, & \alpha_{1}({y_{0}^{4}})={t_{4}^{0}} f_{1}^{-1, 1},\quad \alpha_{2} ({y_{0}^{4}}) = 0 \end{array} $$

and $y_{1} = {y_{0}^{4}}$. We note that *α*_2_(*y*_1_) = 0,

6.2 $$ \alpha_{1} (y_{1}) = -\gamma \delta, \qquad \gamma = \frac{f_{1}^{-1, 1}}{f_{2}^{-1, 1}} \cdot \frac{ f_{2}^{1, 1}}{ f_{1}^{1, 1}}\cdot \frac{f_{1}^{1, -1}}{f_{2}^{1, -1}} \cdot\frac{ f_{2}^{-1, -1}}{ f_{1}^{-1, -1}} $$

and the advanced time $t^{0} = {t_{1}^{0}} + {t_{2}^{0}} + {t_{3}^{0}} + {t_{4}^{0}}$ satisfies

6.3 $$ t^{0} = \kappa \delta, \qquad \kappa = \frac 1{f_{1}^{-1, -1} }\biggl(1 - \frac{f_{2}^{-1, -1}}{f_{2}^{1, -1}} \biggl(1 - \frac{f_{1}^{1, -1}}{f_{1}^{1, 1}}\biggl(1 - \frac{f_{2}^{1, 1}}{f_{2}^{1, -1}}\biggr)\biggr)\biggr) . $$

We are now in exactly the same situation as before with the exception that *δ* is replaced by *γδ* (note that *γ* < 1). We denote by *y*_2_,*y*_3_,… the solution approximations after the next rounds and by *t*^1^,*t*^2^,… the time needed to advance the round. In every round we get an additional factor *γ*. This shows a geometric decay for *α*_1_(*y*_*j*_) → 0, and the total time until convergence is *t*^0^ + *t*^1^ + *t*^2^ + … = *κδ*(1 + *γ* + *γ*^2^ + …) = *κδ*/(1 − *γ*).

Based on this analysis we propose the following algorithm: as soon as we detect the situation of Section 4.4 we stop the integration at a point *y*_0_, where *α*_1_(*y*_0_) = −*δ* with 0 < *δ* ≪ 1, and *α*_2_(*y*_0_) = 0. We then advance the current time by *κδ*/(1 − *γ*) to get *t*^∗^, and we take as solution approximation the vector

6.4 $$ y^{*} = y_{0} + t_{1}^{*} f^{-1, -1} + t_{2}^{*} f^{1, -1} + t_{3}^{*} f^{1, 1} + t_{4}^{*} f^{-1, 1} , $$

where $t_{j}^{*} = {t_{j}^{0}} /(1-\gamma )$. A projection of *y*^∗^ onto *Σ*_1_ ∩*Σ*_2_ is recommended. With this first order analysis we get an error proportional to *δ*^2^. It is therefore reasonable to choose $\delta = \sqrt {\textit {tol}} $, where *tol* is the accuracy required in the integration of the differential equations.

The situation where the solution spirals outwards from a codimension-2 sliding (Fig. 9) can be treated similarly. Here, we have *α*_1_(*y*_0_) = 0 and *α*_2_(*y*_0_) = 0, and without loss of generality we assume (4.14), but this time we have *γ* > 1. We consider

6.5 $$ y^{*} = y_{0} + {t_{1}^{0}} f^{-1, -1} + {t_{2}^{0}} f^{1, -1} + {t_{3}^{0}} f^{1, 1} + {t_{4}^{0}} f^{-1, 1} , $$

which is formally the same as (6.4), but now *y*_0_ ∈*Σ*_1_ ∩*Σ*_2_, *y*^∗^ is the solution approximation after time *κδ*, and the vector field spirals outwards. Up to first order in *δ* (assuming *α*_1_(*y*) and *α*_2_(*y*) to be affine functions) this approximation satisfies *α*_2_(*y*^∗^) = 0 and *α*_1_(*y*^∗^) = −*δ*(*γ* − 1), and can be interpreted as a solution with negative time starting at *y*^∗^ and ending up (after infinitely many rounds) at *y*_0_. With the value *y*^∗^ from (6.5) we can then continue the integration of the outwards spiraling solution. Since *δ* > 0 is a free parameter, we get in this way a one-parameter family of solution approximations.

### *Remark 6.1*

The switching between codimension-2 sliding and (outwards) spiraling solution is related to the bilinear interpolation (2.2) through the condition (4.12). A similar switching has been studied in [11], where instead of bilinear interpolation the author considers moments sliding vector fields, which constitutes of a different kind of Filippov vector fields.

## Conclusion

We have presented an algorithm for the numerical treatment of discontinuous dynamical systems. It considers all generic situations up to codimension two. The main focus is on the switching between different types of solutions (classical and sliding in codimension 1 and 2).

In the case of non-uniqueness of Filippov solutions the algorithm selects the solution that can be interpreted as the limit solution of a regularized differential equation. When exiting a codimension-2 discontinuity hyper-surface, we use a scaling-invariant criterion, which makes the exit point from a codimension-2 sliding unique. In most situations this provides a unique switching. There are three exceptions.

- Entering the intersection *Σ*_1_ ∩*Σ*_2_. In the left two situations of Fig. 6 we have non-uniqueness and the limit solution of a regularization depends on the scaling (see Example 3 of [17]). Putting more weight to one of the constraints permits to select a specific solution.
- Exiting the intersection *Σ*_1_ ∩*Σ*_2_. In the right pictures of Fig. 12 we also have non-uniqueness. This is independent of the scaling, because in any case the basin of attraction of both solutions are non empty (Fig. 13).
- Exiting *Σ*_1_ ∩*Σ*_2_ through spiraling. In the upper left picture of Fig. 12 the solution exits a codimension-2 sliding through spiraling. As a consequence of our scaling-invariant criterion (4.12) we have a one-parameter family of exiting solutions. With the usual criterion (4.11), we would have a two-parameter family of exiting solution, because the exit point is not unique.

## Footnotes

1 In some situations this can be rigorously justified by the study of asymptotic expansions in powers of *ε* together with an estimation of the remainder. However, already for the case of two intersecting surfaces, this is a challenging problem and not much is known in general.
